# The Organic Attitude–Behaviour Gap: Behavioural Schemas in Food Choice

**DOI:** 10.3390/foods15213841

**Published:** 2026-10-27

**Authors:** Anton Kolotusha, Sanat Seitov

**Affiliations:** Department of Agricultural Economics, Faculty of Economics, Lomonosov Moscow State University, Moscow 119991, Russia; sanatpan@mail.ru

**Keywords:** organic food, attitude–behaviour gap, behavioural schemas, vignette methodology, consumer psychology

## Abstract

**Background:** The gap between positive attitudes toward organic food and actual purchase behaviour remains a puzzle for consumer research. This study asks why some consumers reject organic products despite favourable attitudes, and whether antagonistic and avoidant schemas are associated with this gap. **Methods:** The sample consisted of 422 adults who completed 20 forced-choice vignettes about organic food scenarios, together with the Pathological Narcissism Inventory (PNI-28), Generalized Anxiety Disorder scale (GAD-7), and Social Desirability Scale (SDS-16). A behavioural domain of the VEGA instrument captured narcissistic schemas. **Results:** The domain showed moderate internal consistency (*α* = 0.61, *ω* = 0.63) and a two-factor structure (KMO = 0.73, *p* < 0.001). Antagonistic schemas were linked to rejection of organic products (*r* = 0.28, *p* < 0.001), and criterion validity was supported through behavioural decision-making vignettes (VN-6) (*r* = 0.28, *p* < 0.001, *n* = 360). Social desirability was not associated with the domain (*r* = 0.04, *p* = 0.43). Confirmatory factor analysis indicated good absolute fit (RMSEA = 0.039; χ^2^*/df* = 1.65) and near-acceptable incremental fit (CFI = 0.83; TLI = 0.81). **Conclusions:** Behavioural schemas appear to be associated with organic purchases, though the VEGA domain requires further refinement.

## 1. Introduction

Organic food consumption occupies an uncomfortable position in consumer research. Surveys consistently show that consumers value organic products, endorse their environmental and health benefits, and express willingness to buy them [1,2]. Yet actual purchase rates remain low, and the gap between stated attitudes and observed behaviour has proven stubborn [3,4,5]. This attitude–behaviour gap is not a marginal anomaly: it constrains the growth of organic food systems and limits the environmental gains that organic production is meant to deliver.

Most explanations of the gap focus on the actor’s perspective—attitudes, values, and perceived behavioural control [6]. These factors matter, but they leave a substantial portion of the variance unexplained. Two consumers with identical positive attitudes toward organic food may behave in opposite ways: one buys organic regularly, the other avoids it entirely. This divergence suggests that something beyond attitudes is at work—something that shapes how individuals interpret organic products and their own likely behaviour in food-choice situations.

One candidate explanation lies in cognitive schemas—stable patterns of interpretation that shape how individuals read situations before deliberate reasoning takes over [2]. Schemas operate beneath conscious evaluation and guide what a person notices, what they expect, and how they respond. In food choice, schemas may determine whether an organic product is perceived as a reasonable option, an unnecessary expense, or a threat to one’s social standing.

Among the schema-level constructs studied in personality research, narcissistic schemas are particularly relevant to food choices that carry moral and status connotations. Narcissism captures individual differences in entitlement, self-enhancement, and sensitivity to social judgment—features that shape how people respond to products framed as virtuous or responsible. In contrast to broader constructs such as the Dark Triad or status consumption [7], narcissism taps specific schema-level processes that shape how individuals interpret morally charged situations. The Narcissism Spectrum Model differentiates two related yet distinct dimensions: grandiose narcissism, marked by overt self-enhancement and devaluation of others, and vulnerable narcissism, marked by hypersensitivity to criticism and defensive withdrawal [8]. Both dimensions share a common core of entitled self-importance but manifest it differently in consumer behaviour [9]. Grandiose narcissists may dismiss organic products as inferior—”It’s just a marketing scam”—thereby sustaining their sense of superiority over those who “fall for” such appeals [10,11,12]. Vulnerable narcissists, by contrast, may experience social comparison anxiety—”What if they think I can’t afford it?”—which can lead to avoidance or defensive justifications [13]. Schema-level research supports this divergence: narcissism is consistently linked to early maladaptive schemas that shape how individuals interpret social situations [2].

The moral dimension of organic consumption complicates this picture further. Buying organic is no longer a purely practical decision: it has become associated with moral virtue and social responsibility [14]. For some consumers, this moral signal is appealing; for others, it is threatening. A person who buys organic may be seen as virtuous—or as pretentious, depending on who is watching. This makes organic food a particularly charged domain, where a simple purchase can carry implications for self-image and social standing.

Work on moral emotions indicates that this moral signal can trigger defensive reactions. The “do-gooder derogation” effect—the tendency to disparage those who display conspicuous moral virtue—may be particularly pronounced among individuals whose self-esteem hinges on maintaining superiority over others [15]. In the context of organic food, such reactions are not abstract: they translate directly into rejection or avoidance of products that carry a moral signal.

Before turning to the empirical study, it is worth clarifying why narcissism, rather than other personality constructs, is the appropriate lens for this question. Status consumption centres on the acquisition of high-prestige goods, whereas organic food is typically marketed as a moral, rather than a status, signal. Moral identity, although relevant to ethical consumption, does not address the defensive and self-protective mechanisms that characterise narcissistic schemas. Narcissism, in contrast, encompasses both the grandiose tendency to devalue moral signals and the vulnerable tendency to avoid them out of fear of judgment, making it especially well suited to understanding divergent responses to organic food.

These considerations lead to a schema-based account of the organic attitude–behaviour gap. In this account, narcissistic schemas—not attitudes—determine how consumers respond to organic products. Two pathways are possible: one organised around active rejection, the other around passive avoidance. Both would reduce organic purchases, but for different reasons—and this distinction is what a schema-level measure should be able to detect.

Measuring these schemas in the context of organic food choice poses a specific methodological challenge. Standard Likert-type questionnaires are susceptible to social desirability bias: respondents are hesitant to acknowledge entitled or contemptuous thoughts, especially when the topic carries moral weight [16,17]. This issue is particularly acute in organic consumption, where moral judgements are salient and self-presentation concerns are strong.

Forced-choice vignettes provide an alternative. Participants read scenarios and select between competing responses, which lowers self-presentation pressure and taps schema-driven decisions [18,19]. Unlike Likert scales, on which respondents can adjust their level of agreement, forced-choice formats require commitment to one interpretation or action, rendering them more sensitive to underlying schemas. In the present study, this format is used to capture how consumers respond to organic food scenarios and to detect the schemas that shape behaviour.

To test this account, we need a measure that can detect schemas in organic food choice. This article introduces and evaluates such a measure: the behavioural domain of the VEGA instrument (Vignette-based Evaluation of Grandiose and Avoidant schemas), a forced-choice vignette tool developed to capture narcissistic schemas in organic food scenarios. The domain consists of 20 vignettes depicting everyday situations involving organic products, in which respondents choose between two responses: one reflecting an antagonistic (grandiose) schema and the other reflecting an avoidant (vulnerable) schema. The VEGA behavioural domain was administered together with the Pathological Narcissism Inventory (PNI-28), the Generalized Anxiety Disorder scale (GAD-7), the Social Desirability Scale (SDS-16), and a criterion measure of behavioural narcissism in decision-making vignettes (VN-6) in a sample of 422 participants.

Three contributions follow from this work. First, it offers a schema-based account of the organic attitude–behaviour gap, shifting attention from attitudes to the interpretive patterns that shape behaviour. Second, it introduces a new forced-choice measure for detecting these patterns in organic food choice. Third, it provides preliminary evidence that narcissistic schemas are systematically linked to organic food decisions, while identifying items that call for refinement.

Three hypotheses were tested, focusing on associations rather than causal or incremental effects:

H1: Antagonistic schemas are positively associated with rejection of organic food.
H2: Avoidant schemas are positively associated with anxiety and avoidance in organic food choice.
H3: Narcissistic schemas are positively associated with self-reported behavioural responses in organic food scenarios.

Because this is an initial test of a new measure, all hypotheses were treated as exploratory rather than confirmatory. To assess the plausibility of the hypothesised two-factor structure of the VEGA behavioural domain, exploratory and confirmatory factor analyses were also performed. Given the exploratory character of the study, the instrument should be regarded as a preliminary version that requires further validation. All findings should be interpreted with this limitation in mind.

## 2. Materials and Methods

### 2.1. Participants and Procedure

Participants were recruited through Prolific (www.prolific.co, accessed on 31 August 2026) and completed an online survey in August 2026. Eligibility criteria were: age 18 or above, fluency in English, and no previous participation in similar research on food choice. The target sample size was set before data collection, following the common heuristic of roughly ten respondents per item for factor analysis [20]. The analytical sample consisted of 422 adults.

Table 1 summarises demographic characteristics and descriptive statistics for the key variables.

The sample was 49.3% female (*n* = 208), with a mean age of 42.2 years (*SD* = 13.5, *n* = 417). Most respondents (43.4%) held a bachelor’s degree, and 61.4% had completed at least a bachelor’s degree. Full-time employment was the most common status (53.1%), followed by part-time (14.0%) and self-employed or freelance work (10.0%). The largest share of participants lived in the United Kingdom (46.2%) or the United States (14.5%), with smaller groups in Canada (8.1%) and Australia (4.0%). The sample was therefore predominantly Western, educated, and English-speaking; this limits generalisability, as discussed in Section 4.6.

The VN-6 criterion measure was collected in a supplementary wave. Of the 422 participants, 360 completed this wave; the remaining 62 did not and were excluded from criterion validity analyses. Completers and non-completers are compared in Section 3. All respondents gave informed consent before taking part. Attention checks were included in the survey; no participant failed more than one, so no cases were excluded.

### 2.2. Design

The study used a cross-sectional survey design. Respondents completed the instruments in a fixed sequence: (1) the VEGA behavioural vignettes, (2) the Pathological Narcissism Inventory (PNI-28), (3) the Generalized Anxiety Disorder scale (GAD-7), (4) the Social Desirability Scale (SDS-16), and (5) sociodemographic items. Placing the vignettes first was intended to reduce the influence of self-report priming on schema-driven responses [18,21]. The fixed order, however, may introduce order effects, and future work should consider counterbalancing. Completion time was approximately 20–25 min.

### 2.3. Measures

*VEGA—Behavioural Domain*. The behavioural domain of the VEGA instrument (Vignette-based Evaluation of Grandiose and Avoidant schemas) consists of 20 forced-choice vignettes. Each vignette presents an everyday situation involving organic food—for example, choosing between organic and conventional products, responding to social pressure about food choices, or reacting to price differences. Respondents select one of two responses, each representing a different interpretation of their own likely behaviour.

In every vignette, one option reflects an antagonistic (grandiose) schema and the other an avoidant (vulnerable) schema. Items 1–10 were designed to capture antagonistic responses; items 11–20 were designed to capture avoidant responses. Antagonistic responses express grandiosity, entitlement, devaluation, and rejection of socially desirable behaviour—for instance, dismissing organic products as “overpriced hype” or “a marketing scam for naive people.” These items draw on the conceptualisation of grandiose narcissism as involving derogation of others and attribution of negative motives to socially desirable behaviour [8,11]. Avoidant responses express anxiety, upward social comparison, self-criticism, and withdrawal—for example, anxiety about being judged for buying conventional products or fear of appearing pretentious for buying organic. These items reflect the conceptualisation of vulnerable narcissism as involving hypersensitivity to social comparison and fear of negative evaluation [13,22].

Responses were scored dichotomously (1 = narcissistic response, 0 = neutral or healthy). To reduce response set bias, the narcissistic option was counterbalanced across items (10 items with option A as narcissistic, 10 items with option B as narcissistic). Total scores range from 0 to 20, with higher values indicating stronger endorsement of narcissistic schemas in organic food choice. In the present sample, observed scores ranged from 0 to 18. The full vignette texts appear in Appendix A. During instrument development, some avoidant items were refined so that the healthy option was clearly distinguishable from the avoidant option. These refinements did not affect data coding or analysis. The original formulations are available on request.

*Pathological Narcissism Inventory (PNI-28)*. Trait pathological narcissism was assessed with the 28-item short form of the PNI [22]. Respondents rated each item on a 7-point scale (1 = strongly disagree, 7 = strongly agree). Cronbach’s alpha in the present sample was 0.93.

*Generalized Anxiety Disorder Scale (GAD-7).* Anxiety symptoms over the preceding two weeks were measured with the GAD-7 [23]. Items were rated on a 4-point scale. Cronbach’s alpha was 0.92.

*Social Desirability Scale (SDS-16).* Social desirability bias was captured with the 16-item version of the Social Desirability Scale-17 (SDS-17; hereafter SDS-16) [24]. As in the original validation study [24], Item 4 (“I have tried illegal drugs”) was omitted from total scores because of its low item-total correlation in the original validation sample. Cronbach’s alpha was 0.77.

*Behavioural Narcissism Vignettes (VN-6).* As a criterion measure, respondents completed six decision-making vignettes assessing narcissistic behavioural tendencies in work-related situations (for example, responding to public criticism, admitting mistakes, or choosing between competence and loyalty when hiring). The VN-6 was developed for this study as an independent criterion measure. Items were adapted from established decision-making paradigms and reviewed for face validity by two researchers. Responses were scored dichotomously (1 = narcissistic choice, 0 = healthy choice). Total scores range from 0 to 6. The full VN-6 vignettes appear in Appendix B. VN-6 data were available for 360 of the 422 participants (85.3%).

*Demographic Variables*. Sociodemographic items covered age, gender, education, employment status, country of residence, native language, English proficiency, and any prior experience with agriculture or farming.

### 2.4. Attention Checks

Three attention checks were included in the survey to monitor data quality [25]. Respondents were asked either to choose a designated response option (for example, “Please select option B for this item”) or to answer a simple factual question (for example, “What is the capital of France?”). No respondent failed more than one check, so no cases were excluded on this basis.

### 2.5. Analytical Strategy

Analyses were performed in Python 3.13 using pandas, numpy, scipy, factor_analyzer, semopy, and statsmodels. Descriptive statistics, reliability estimates, and Pearson correlations were computed for all key variables.

*Descriptive Statistics.* Means, standard deviations, and ranges were computed for the VEGA behavioural domain, PNI-28, GAD-7, SDS-16, and VN-6.

*Reliability.* Internal consistency was evaluated with Cronbach’s alpha and McDonald’s omega. Because the vignette items are binary, alpha values between 0.60 and 0.70 were treated as acceptable for exploratory work [26].

*Item Analysis.* For each of the 20 VEGA items, item difficulty, item-total correlations, and alpha-if-item-deleted were examined to identify poorly performing items.

**Figure 1:**
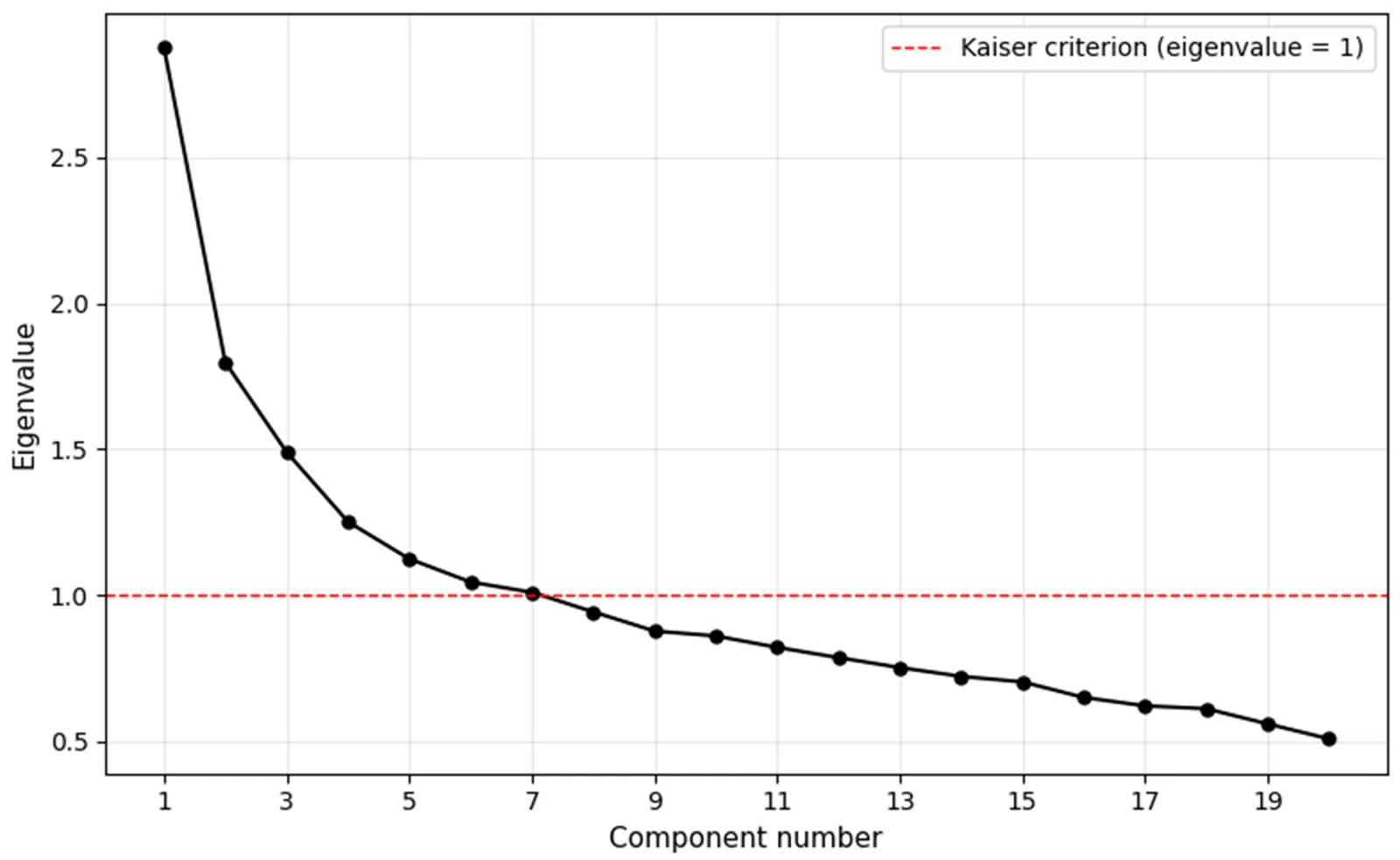
Scree plot for the VEGA behavioural domain (*N* = 422). The plot shows eigenvalues for the first 20 components. The clear break after the second component supports the two-factor solution.

*Exploratory Factor Analysis (EFA).* To examine the hypothesised structure, an exploratory factor analysis with varimax rotation was performed, specifying a two-factor solution based on theoretical predictions (antagonistic and avoidant schemas). Data suitability was assessed using the Kaiser–Meyer–Olkin (KMO) measure (values above 0.70 are considered acceptable, and above 0.80 good [27]) and Bartlett’s test of sphericity [28]. Factor loadings of 0.40 or above were treated as acceptable. Communalities were also inspected. The scree plot was examined to determine the number of factors to retain (Figure 1). Although the Kaiser criterion suggested a larger number of components, the scree plot and theoretical considerations supported a two-factor solution.

*Confirmatory Factor Analysis (CFA).* A confirmatory factor analysis (CFA) was performed using maximum likelihood estimation with weighted adjustments (MLW) to test the hypothesised two-factor structure, with items 1–10 assigned to the Antagonistic factor and items 11–20 assigned to the Avoidant factor. Model fit was evaluated using CFI, TLI, RMSEA, and χ^2^/df. Because the items are binary, MLW served as an approximation; WLSMV estimation with a larger sample is recommended for future validation (see Appendix C).

*Convergent Validity*. Convergent validity was assessed through Pearson correlations between the VEGA behavioural domain and the PNI-28 total score.

*Discriminant Validity*. Discriminant validity was examined through Pearson correlations between the VEGA behavioural domain and the SDS-16.

*Criterion Validity*. Criterion validity was assessed through Pearson correlations between the VEGA behavioural domain and the VN-6 total score.

*Subgroup Analyses*. Subgroup correlations were computed for gender, age, education, anxiety, and PNI level. To control for multiple comparisons, *p*-values were adjusted using the Benjamini–Hochberg false discovery rate (FDR) procedure [29].

*Acquiescence Bias.* Acquiescence bias was assessed by examining the mean proportion of narcissistic responses across items.

*Correlation Matrix.* A full Pearson correlation matrix was computed among all key variables.

### 2.6. Ethics Statement

The study collected anonymous survey data and posed minimal risk to respondents. Lomonosov Moscow State University does not have a dedicated ethics committee for non-interventional social science research involving anonymous surveys. Because the research involved no sensitive personal data, no vulnerable populations, and no interventions, formal ethical review was not required. All procedures were conducted in accordance with the principles of the 1964 Helsinki Declaration and its later amendments. Every respondent gave informed consent before taking part. Respondents were informed about the purpose of the study, the voluntary nature of participation, and their right to withdraw at any time without penalty. Data were collected anonymously, and no personally identifiable information was recorded.

## 3. Results

### 3.1. Descriptive Statistics

Table 1 reports descriptive statistics for the main variables. The VEGA behavioural domain had a mean of 4.47 (*SD* = 2.71, range 0–18). The PNI-28 total averaged 97.65 (*SD* = 27.60). The GAD-7 mean was 5.12 (*SD* = 5.11), below the clinical cutoff of 10. The SDS-16 averaged 6.86 (*SD* = 3.52). The VN-6 mean was 0.96 (*SD* = 1.19, range 0–6, *n* = 360).

### 3.2. Completers vs. Non-Completers

A comparison of VN-6 completers (*n* = 360) and non-completers (*n* = 62) showed significant differences for education (*p* = 0.002), PNI-28 (*p* = 0.019), and GAD-7 (*p* = 0.005), but not for age, gender, VEGA scores, or SDS-16 (all *p* > 0.05). Completers reported a higher mean education level (*M* = 2.75 vs. *M* = 2.14), lower PNI-28 scores (*M* = 96.91 vs. *M* = 109.34), and lower GAD-7 scores (*M* = 4.86 vs. *M* = 7.59) than non-completers. The VN-6 subsample was therefore slightly more educated and less anxious than the full sample. However, given the unequal sample sizes (360 vs. 62), this comparison should be interpreted cautiously.

### 3.3. Item Analysis

Item difficulty ranged from 0.03 (VEGA_2) to 0.52 (VEGA_1 and VEGA_3), with a mean of 0.22. Item-total correlations ranged from 0.03 (VEGA_3) to 0.40 (VEGA_16). Eight items had item-total correlations below 0.20, indicating weak discrimination. As noted in Section 3.5, removing any single item did not raise alpha above 0.63, which suggests that the low internal consistency reflects the heterogeneous content of the vignettes rather than specific problematic items. The mean proportion of narcissistic responses was 0.22 (*SD* = 0.14), below the 0.50 expected by chance, indicating that respondents did not display a systematic acquiescence bias.

### 3.4. Factor Structure

An exploratory factor analysis with varimax rotation was performed on the 20 VEGA behavioural vignette items. The Kaiser–Meyer–Olkin (KMO) measure of sampling adequacy was 0.73, and Bartlett’s test of sphericity was significant, χ^2^(190) = 814.2, *p* < 0.001, indicating that the data were suitable for factor analysis. The first five eigenvalues were 2.88, 1.80, 1.49, 1.25, and 1.12. Although the Kaiser criterion (eigenvalue > 1) suggested up to seven components [30], the scree plot (Figure 1) showed a clear break after the second factor, supporting a two-factor solution.

As shown in Table 2,six items loaded strongly (≥0.40) on Factor 1 (Antagonistic Schema) and three items on Factor 2 (Avoidant Schema). Items loading on Factor 1 reflected dismissal of organic products as overpriced, distrust of organic labelling, and rejection of organic consumption as a status signal. Items loading on Factor 2 reflected social anxiety, fear of judgment, and self-consciousness about purchasing organic products. Communalities ranged from 0.01 (VEGA_3) to 0.42 (VEGA_16), with a mean of 0.16, indicating that the two-factor solution left a substantial proportion of item variance unexplained.

The two-factor solution explained 23.4% of the total variance (Factor 1: 14.4%; Factor 2: 9.0%). This relatively low proportion is consistent with the heterogeneous content of the forced-choice vignettes, which sample diverse behavioural contexts.

**Table 2:** Factor loadings for the VEGA behavioural domain (varimax rotation, two-factor solution).

Item	Factor 1 (Antagonistic)	Factor 2 (Avoidant)	*h* ^2^
VEGA_1	0.02	0.21	0.05
VEGA_2	0.05	0.26	0.07
VEGA_3	0.01	0.08	0.01
VEGA_4	0.08	**0.45**	0.21
VEGA_5	0.16	−0.01	0.03
VEGA_6	0.19	0.04	0.04
VEGA_7	0.12	**0.54**	0.31
VEGA_8	0.21	−0.01	0.04
VEGA_9	−0.06	0.35	0.13
VEGA_10	0.17	**0.57**	0.36
VEGA_11	0.25	−0.04	0.06
VEGA_12	0.28	−0.05	0.08
VEGA_13	0.23	0.02	0.06
VEGA_14	**0.42**	0.08	0.18
VEGA_15	**0.47**	0.10	0.24
VEGA_16	**0.64**	−0.07	0.42
VEGA_17	**0.41**	0.05	0.17
VEGA_18	0.35	−0.12	0.14
VEGA_19	**0.54**	0.11	0.30
VEGA_20	**0.53**	0.03	0.29

Note. *N* = 422. Loadings ≥ 0.40 are shown in **bold**. *h*^2^ = communality. KMO = 0.73; Bartlett’s test χ^2^(190) = 814.2, *p* < 0.001. Variance explained: Factor 1 = 14.4%, Factor 2 = 9.0% (total 23.4%). Mean communality = 0.16.

A confirmatory factor analysis (CFA) was then performed, with items 1–10 assigned to the Antagonistic factor and items 11–20 assigned to the Avoidant factor. The model showed good absolute fit (RMSEA = 0.039; χ^2^*/df* = 1.65) and near-acceptable incremental fit (CFI = 0.83; TLI = 0.81). Standardised loadings ranged from 0.08 (VEGA_5) to 0.59 (VEGA_10). Three items on the Antagonistic factor (VEGA_4, VEGA_7, VEGA_10) and six items on the Avoidant factor (VEGA_14, VEGA_15, VEGA_16, VEGA_17, VEGA_19, VEGA_20) had loadings above 0.40. These results suggest that while the general two-factor structure is plausible and broadly consistent with the data, the Antagonistic factor requires further refinement. Full CFA results are reported in Appendix C.

### 3.5. Reliability

Internal consistency for the VEGA behavioural domain was *α* = 0.61 (*ω* = 0.63; Table 1). This falls below the conventional threshold of 0.70 but remains within the range considered acceptable for exploratory work with binary forced-choice items [19]. Item analysis showed that eight of the 20 items had item-total correlations below 0.20, and communalities ranged from 0.01 to 0.42 (mean = 0.16). Removing any single item did not raise alpha above 0.63, which suggests that the low internal consistency reflects the heterogeneous content of the vignettes rather than specific problematic items. The PNI-28 (*α* = 0.93), GAD-7 (*α* = 0.92), and SDS-16 (*α* = 0.77) showed acceptable reliability.

### 3.6. Convergent and Discriminant Validity

The VEGA behavioural domain correlated significantly with the PNI-28 total score (*r* = 0.28, *p* < 0.001), indicating that organic-specific narcissistic schemas are associated with trait pathological narcissism. This supports the convergent validity of the instrument. Discriminant validity was examined through the correlation between the VEGA behavioural domain and the SDS-16. This correlation was non-significant and near zero (*r* = 0.04, *p* = 0.43), indicating that the instrument is not contaminated by social desirability bias.

These correlations appear in Table 3. The VEGA domain correlated significantly with the PNI-28 total and with VN-6, supporting convergent and criterion validity, while remaining uncorrelated with the SDS-16 and the GAD-7, supporting discriminant validity.

The full correlation matrix among all key variables appears in Table 4, which also includes inter-scale correlations.

The full matrix (Table 4) shows that the PNI-28 correlated significantly with the GAD-7 (*r* = 0.35, *p* < 0.001) and the SDS-16 (*r* = 0.21, *p* < 0.001), consistent with prior work on the overlap between pathological narcissism, anxiety, and social desirability. The VEGA domain, by contrast, was uncorrelated with the SDS-16 (*r* = 0.04, *p* = 0.430) and the GAD-7 (*r* = 0.05, *p* = 0.285), supporting its discriminant validity and indicating that the instrument captures narcissistic schemas rather than general negative affect.

### 3.7. Criterion-Related Evidence

The association between the VEGA behavioural domain and the VN-6 behavioural decision-making vignettes was examined next. The correlation was significant (*r* = 0.28, *p* < 0.001, *n* = 360), indicating that organic-specific narcissistic schemas generalise to narcissistic decision-making in work-related scenarios. This supports criterion validity. Because both VEGA and VN-6 use a forced-choice vignette format, however, the observed correlation may partly reflect shared method variance. Future studies should validate VEGA against non-vignette criterion measures (for example, behavioural observations, informant reports, or real-world purchase data).

### 3.8. Subgroup Analyses

To explore criterion validity further, subgroup analyses were conducted across gender, age, education, anxiety, and narcissism levels. To control for multiple comparisons, *p*-values were adjusted using the Benjamini–Hochberg false discovery rate (FDR) procedure. The VEGA behavioural domain correlated significantly with VN-6 in all ten subgroups after FDR correction (Table 5): women (*r* = 0.33, *p*_FDR < 0.001), men (*r* = 0.23, *p*_FDR = 0.002), younger adults (<40 years; *r* = 0.34, *p*_FDR < 0.001), older adults (≥40 years; *r* = 0.25, *p*_FDR < 0.001), respondents with higher education (*r* = 0.26, *p*_FDR < 0.001), respondents without higher education (*r* = 0.32, *p*_FDR < 0.001), low anxiety (*r* = 0.34, *p*_FDR < 0.001), high anxiety (*r* = 0.21, *p*_FDR = 0.005), low PNI (*r* = 0.26, *p*_FDR < 0.001), and high PNI (*r* = 0.25, *p*_FDR = 0.001).

The subgroup correlations, while statistically significant, were modest in magnitude (*r* = 0.21–0.34) and should be interpreted as exploratory. They suggest consistent associations but do not establish measurement equivalence across groups.

## 4. Discussion

This study examined whether narcissistic schemas help explain the organic attitude–behaviour gap. The findings offer preliminary support for a schema-based account: the VEGA behavioural domain captured two distinct patterns—antagonistic and avoidant—and these patterns were associated with trait narcissism and with behavioural decision-making. At the same time, the psychometric evidence indicates that the instrument requires further refinement before it can be used as a standalone measure.

### 4.1. Factor Structure and Reliability

Exploratory factor analysis broadly supported the two-factor structure (antagonistic and avoidant schemas). Six items loaded strongly on the antagonistic factor, reflecting devaluation, distrust, and dismissal of organic consumption, and three items loaded on the avoidant factor, reflecting social anxiety and fear of judgment. This structure aligns with the Narcissism Spectrum Model, which distinguishes between grandiose and vulnerable expressions of narcissism [8].

As reported in Section 3.4, the CFA showed good absolute fit and near-acceptable incremental fit. Several items, however, had low standardised loadings (for example, VEGA_5 = 0.08, VEGA_3 = 0.09, VEGA_8 = 0.10), suggesting that the two-factor structure is plausible and broadly consistent with the data, but that the Antagonistic factor requires refinement. This pattern is consistent with the heterogeneous content of the forced-choice vignettes, which sample diverse behavioural contexts. It should be noted, however, that the EFA-identified factors did not perfectly align with the theoretical assignment of items to antagonistic and avoidant schemas. Three items designed to capture antagonistic responses (VEGA_4, VEGA_7, VEGA_10) loaded on the Avoidant factor, while six items designed to capture avoidant responses (VEGA_14–VEGA_17, VEGA_19, VEGA_20) loaded on the Antagonistic factor. This pattern may reflect the fact that both schema types share a common core of entitled self-importance [8], and that behavioural manifestations of antagonistic and avoidant schemas can overlap in food choice contexts. For example, dismissing organic products as “overpriced” (designed as antagonistic) may also serve an avoidant function by reducing anxiety about social comparison. Conversely, anxiety about being judged for buying organic (designed as avoidant) may also involve antagonistic elements, such as resentment toward those who judge.

These findings align with the Narcissism Spectrum Model [8], which posits that grandiose and vulnerable narcissism share a common core of entitled self-importance. The overlap between antagonistic and avoidant responses observed here is consistent with Morf and Rhodewalt’s [11] self-regulatory processing model, which emphasizes that narcissistic behaviours can serve both self-enhancement and defensive functions. This overlap may explain why some items designed to capture one schema loaded on the other.

Future iterations of the instrument should refine items to differentiate the two schema types more clearly.

Internal consistency was moderate (Section 3.5): below the conventional 0.70 threshold, yet within the range considered acceptable for exploratory work with binary forced-choice items. Forced-choice vignettes deliberately introduce heterogeneity by presenting different situational contexts, which can suppress alpha while preserving construct validity [19]. As reported in Section 3.5, removing any single item did not raise alpha above 0.63, indicating that the low internal consistency reflects the instrument’s broad content coverage rather than specific problematic items. The reliability estimate nonetheless suggests that further refinement of the vignette items is needed.

### 4.2. Convergent Validity

Convergent validity with trait narcissism was supported. The VEGA behavioural domain correlated significantly with the PNI-28 total score (Section 3.6), indicating that organic-specific narcissistic schemas are meaningfully associated with pathological narcissism. This is notable given the domain-specific nature of the vignettes: while the PNI-28 measures global trait narcissism, the VEGA behavioural domain captures how narcissistic schemas manifest in food choice contexts specifically. The moderate correlation (*r* = 0.28) suggests that the instrument measures related but not identical constructs—which is theoretically expected for a domain-specific measure. The PNI-28, however, was designed for clinical and subclinical populations, and its sensitivity to subclinical variation in a general-population sample may be limited. Future studies should include alternative measures of subclinical narcissism (for example, NPI-13 or NARQ) to provide a more comprehensive assessment of convergent validity. The modest correlation (r = 0.28) is consistent with previous work [9,10] suggesting that the VEGA captures a contextualized expression of narcissism that is not fully reducible to global trait measures.

### 4.3. Discriminant Validity

The VEGA behavioural domain was unrelated to social desirability (Section 3.6), indicating that the forced-choice format reduces self-presentation bias effectively. This is a key advantage of vignette methodology over Likert-type scales, on which respondents can adjust their answers to appear more socially desirable [16,18]. The absence of social desirability contamination suggests that respondents were willing to endorse antagonistic or avoidant responses without concern for how they would be perceived.

### 4.4. Criterion Validity

The VEGA behavioural domain correlated significantly with the VN-6 behavioural decision-making vignettes (Section 3.7). This indicates that organic-specific narcissistic schemas generalise to narcissistic decision-making in other domains, such as responses to criticism, willingness to admit mistakes, and preferences for competence versus loyalty in hiring. The magnitude of this correlation (Section 3.7) is typical for cross-domain associations in personality research and provides evidence that the VEGA instrument captures a generalisable narcissistic tendency rather than purely context-specific responses. Future studies should validate VEGA against non-vignette criterion measures, such as behavioural observations, informant reports, or real-world purchase data (see Section 4.6).

Subgroup analyses with FDR correction further supported the robustness of criterion validity. The VEGA behavioural domain correlated significantly with the VN-6 across all ten demographic and psychological subgroups examined, including gender, age, education, anxiety level, and PNI level. This consistency indicates that the instrument performs similarly across diverse population segments and is not driven by any particular subgroup.

### 4.5. Implications for Food Choice Research

These findings carry preliminary implications for understanding organic food consumption. The VEGA behavioural domain captures antagonistic schemas (dismissal of organic as overpriced hype) and avoidant schemas (anxiety about social judgment), both of which may act as barriers to organic consumption. Traditional attitude measures do not capture these schemas, because they focus on conscious evaluations rather than underlying personality-driven predispositions. While these findings require validation against real-world purchase behaviour before applied recommendations can be made, while these findings require validation, they tentatively point to a direction for future research: testing whether different messaging strategies are effective for consumers with different schema profiles. Consumers with elevated antagonistic schemas may respond to messages that address their scepticism directly, whereas those with avoidant schemas may benefit from social norm interventions that reduce anxiety about judgment.

### 4.6. Limitations and Future Directions

Several limitations should be noted. First, the cross-sectional design precludes causal inferences about the relationship between narcissistic schemas and food choice behaviour. Second, the sample was predominantly Western, educated, and English-speaking, which limits generalisability to other cultural contexts. Third, internal consistency (*α* = 0.61; *ω* = 0.63) fell below conventional standards, and a confirmatory factor analysis showed good absolute fit (Section 3.4) but near-acceptable incremental fit (CFI = 0.83; TLI = 0.81), indicating that the vignette items require further refinement. Future iterations should aim for more homogeneous item content within each schema type or incorporate conflict-based scenarios to increase response variance. Pearson correlations were used for binary vignette items; tetrachoric correlations [31] may provide more accurate estimates for such data and should be considered in future studies. Fourth, criterion validity was established using vignette-based scenarios rather than real-world purchase behaviour; future studies should validate VEGA against actual food purchases or observed consumer behaviour. Fifth, because both VEGA and VN-6 use a forced-choice vignette format, the observed correlation may partly reflect shared method variance; future studies should include non-vignette criterion measures. Sixth, the absence of alternative narcissism measures limits conclusions about whether VEGA captures grandiose narcissism, vulnerable narcissism, or a broader schema-based response style (Section 4.2). Seventh, the present study did not assess incremental validity; future research should examine whether VEGA predicts food choice outcomes above and beyond established measures of narcissism and attitudes. Eighth, the study did not directly measure attitudes toward organic food or actual purchase behaviour. Therefore, H3 was tested as an association, not as an incremental validity test. Future studies should include direct attitude measures to examine whether schemas explain variance beyond attitudes. Ninth, the criterion measure (VN-6) is vignette-based, which may introduce shared method variance with the VEGA instrument; future studies should include non-vignette criterion measures. Finally, the use of an online panel (Prolific) may introduce self-selection bias and limit generalisability to broader consumer populations.

## 5. Conclusions

This study offers preliminary evidence that narcissistic schemas are associated with organic food choice. However, the absence of direct attitude and behavioural measures limits conclusions about their incremental validity. The VEGA behavioural domain showed moderate internal consistency (*α* = 0.61; *ω* = 0.63), a two-factor structure separating antagonistic and avoidant schemas, convergent validity with trait narcissism (*r* = 0.28, *p* < 0.001), discriminant validity from social desirability (*r* = 0.04, *p* = 0.43), and criterion validity with behavioural decision-making vignettes (*r* = 0.28, *p* < 0.001, *n* = 360). A confirmatory factor analysis indicated good absolute fit (RMSEA = 0.039; χ^2^*/df* = 1.65) and near-acceptable incremental fit (CFI = 0.83; TLI = 0.81), showing that the factor structure requires refinement. The forced-choice vignette format appears to capture domain-specific narcissistic schemas in organic food choice without social desirability contamination. The instrument requires further refinement, particularly through conflict-based scenarios, item revision, and validation against real-world purchase behaviour. Future research should also examine whether the instrument predicts organic food choice outcomes above and beyond established measures of narcissism and attitudes.

## Figures and Tables

**Table 1:** Sample characteristics and descriptive statistics for key variables.

Variable	*M*	*SD*	Min	Max	*α*	*ω*
Age (years)	42.21	13.50	22	70	—	—
Gender (% female)	49.3	—	—	—	—	—
Education (% bachelor+)	61.4	—	—	—	—	—
VEGA Behavioural Domain (20 items)	4.47	2.71	0	18	0.61	0.63
PNI-28 Total	97.65	27.60	34	166	0.93	—
GAD-7	5.12	5.11	0	21	0.92	—
SDS-16	6.86	3.52	0	16	0.77	—
VN-6	0.96	1.19	0	6	—	—

Note. *N* = 422 for all variables except VN-6 (*n* = 360) and age (*n* = 417). VEGA = Vignette-based Evaluation of Grandiose and Avoidant schemas; PNI-28 = Pathological Narcissism Inventory, 28-item version; GAD-7 = Generalized Anxiety Disorder, 7-item scale; SDS-16 = Social Desirability Scale, 16-item version; VN-6 = Behavioural narcissism vignettes, 6 items. *α* = Cronbach’s alpha; *ω* = McDonald’s omega.

**Table 3:** Pearson correlations between the VEGA behavioural domain and validity measures.

Variable	*r*	*p*	*n*
PNI-28 total	0.28	<0.001	422
GAD-7	0.05	0.285	422
SDS-16	0.04	0.430	422
VN-6	0.28	<0.001	360

Note. VEGA = Vignette-based Evaluation of Grandiose and Avoidant schemas; PNI-28 = Pathological Narcissism Inventory, 28-item version; GAD-7 = Generalized Anxiety Disorder, 7-item scale; SDS-16 = Social Desirability Scale, 16-item version; VN-6 = Behavioural narcissism vignettes, 6 items. *n* = 422 for PNI-28, GAD-7, SDS-16; *n* = 360 for VN-6 correlations due to the second data collection wave.

**Table 4:** Full Pearson correlation matrix among key variables.

Variable	1	2	3	4	5
1. VEGA behavioural domain	—				
2. PNI-28 total	0.28 ***	—			
3. GAD-7	0.05	0.35 ***	—		
4. SDS-16	0.04	0.21 ***	0.23 ***	—	
5. VN-6	0.28 ***	0.16 **	−0.06	−0.01	—

Note. *N* = 422 for all variables except VN-6 (*n* = 360). VEGA = Vignette-based Evaluation of Grandiose and Avoidant schemas; PNI-28 = Pathological Narcissism Inventory, 28-item version; GAD-7 = Generalized Anxiety Disorder, 7-item scale; SDS-16 = Social Desirability Scale, 16-item version; VN-6 = Behavioural narcissism vignettes, 6 items. ** *p* < 0.01, *** *p* < 0.001.

**Table 5:** Subgroup correlations between VEGA behavioural domain and VN-6.

Subgroup	*n*	*r*	*p*	*p*_FDR
Women	172	0.33	<0.001	<0.001
Men	181	0.23	0.002	0.002
Age < 40	123	0.34	<0.001	<0.001
Age ≥ 40	234	0.25	<0.001	<0.001
Higher education	236	0.26	<0.001	<0.001
No higher education	119	0.32	<0.001	<0.001
Low anxiety	180	0.34	<0.001	<0.001
High anxiety	180	0.21	0.005	0.005
Low PNI	183	0.26	<0.001	<0.001
High PNI	177	0.25	<0.001	0.001

Note. *n* = 360 (VN-6 subsample). Higher education = bachelor’s degree or higher. Low/high anxiety split at GAD-7 = 4. Low/high PNI split at PNI-28 = 99 (median). *p*_FDR = Benjamini–Hochberg false discovery rate-adjusted *p*-value.

## Data Availability

The data presented in this study are available on request from the corresponding author. The raw survey data are not publicly available due to privacy restrictions and the absence of participant consent for public sharing of individual-level responses. The coding script is available from the corresponding author on request.

## Abbreviations

The following abbreviations are used in this manuscript:

VEGA	Vignette-based Evaluation of Grandiose and Avoidant schemas
PNI-28	Pathological Narcissism Inventory, 28-item version
GAD-7	Generalized Anxiety Disorder, 7-item scale
SDS-16	Social Desirability Scale, 16-item version
VN-6	Behavioural narcissism vignettes, 6 items
EFA	Exploratory Factor Analysis
CFA	Confirmatory Factor Analysis
KMO	Kaiser–Meyer–Olkin
FDR	False Discovery Rate
SD	Standard Deviation
M	Mean
MLW	Maximum Likelihood with Weighted adjustments
CFI	Comparative Fit Index
TLI	Tucker–Lewis Index
RMSEA	Root Mean Square Error of Approximation

## Appendix A

**Table A1:** VEGA behavioural vignettes: Organic food choice domain (items 1–20).

Item	Situation	Option A	Option B
VEGA_1	You buy regular milk, even though organic costs only 20% more.	**“I don’t need a label to tell me what’s healthy. I’ve been drinking regular milk for years and I’m fine.”**	“I compared reviews carefully and honestly don’t see any real difference in taste or quality.”
VEGA_2	A friend recommends an expensive organic food store. You refuse to go.	“He’s free to recommend, but I have my own preferences and habits.”	**“He thinks his opinion should matter more than mine. That really irritates me.”**
VEGA_3	At the supermarket, you take regular apples, even though organic ones are right next to them.	**“An organic sticker doesn’t make apples taste better. I’m not paying extra for the exact same thing.”**	“I honestly don’t see the point of paying extra just for a different label on the same fruit.”
VEGA_4	A coworker brags about buying only eco-products. You decide not to follow their example.	“Everyone has their own priorities when shopping. I don’t need to follow hers at all.”	**“She clearly thinks she’s better than me just because she buys eco-products.”**
VEGA_5	You read an article about the dangers of pesticides in conventional vegetables. You decide to keep buying conventional.	**“These articles are just scare tactics. I’ve read enough to know regular food is perfectly safe.”**	“Pesticide levels in regular products are usually within established safety limits and norms.”
VEGA_6	A market vendor insists that his tomatoes are “organic, no chemicals.” You decide not to buy.	“No certificate means no proof. I have no reason to take his word over proper labeling.”	**“If his tomatoes were really organic, he wouldn’t have to push this hard. Something’s off here.”**
VEGA_7	Your friends have switched to organic and are trying to convince you. You stick to your opinion.	“We simply have different eating habits. I don’t see the need to argue about it with them.”	**“I don’t need a diet lecture from them. I’ve thought about this and made my own decision.”**
VEGA_8	You see that organic products cost twice as much. You walk past and take regular ones.	**“Twice the price for a label? No thanks. I eat perfectly well without paying for marketing.”**	“The price difference is simply too big, and I honestly don’t see the extra value in it.”
VEGA_9	At a restaurant, you order a dish made with regular ingredients rather than organic (which is on the menu).	“I’ve tried both versions and honestly the taste difference isn’t nearly enough to justify the extra cost.”	**“I’m not paying extra just because the menu puts ‘organic’ next to something. It’s the same dish.”**
VEGA_10	Your neighbor says, “Only organic is real.” You don’t argue, but you don’t change your habits.	“Arguing about food choices is pointless. I make my own choices, and she makes hers.”	**“She acts like she knows better than me. I don’t need her approval to buy what I want.”**
VEGA_11	You bought organic milk because you were afraid of being judged by a friend who watches her diet. At home you feel you wasted money.	**“I felt so self-conscious about seeming broke. I hate how much I care about her opinion.”**	“Her opinion doesn’t define my worth. Next time I’ll buy what I actually want.”
VEGA_12	You started ordering groceries from an expensive eco-delivery service because acquaintances laughed at you for going to a regular supermarket. You feel you acted foolishly.	“Their laughter says more about them than about me. I’ll shop where I want.”	**“I was so humiliated being seen as out of touch—I paid extra to avoid that.”**
VEGA_13	You didn’t buy organic vegetables because you were afraid your friends would call you a “health nut.” Later you regret it.	**“I’m ashamed that I gave up what I wanted just to avoid being teased.”**	“I don’t need to justify my food choices to anyone. I’ll buy what I prefer.”
VEGA_14	You didn’t buy organic meat because you were embarrassed it would look like “showing off.” At home you think you were worried for nothing.	**“I worried they’d see me as a show-off. I stuck with regular just to avoid that judgment.”**	“People buy what they like. I’ll buy organic next time if that’s what I want.”
VEGA_15	You bought regular tomatoes instead of organic because you didn’t want to look like a “show-off” in front of an acquaintance. Later you regret it.	“I don’t need to worry about seeming pretentious. I’ll buy what I want.”	**“Dreaded her thinking I’m pretentious—so I played it safe and bought regular.”**
VEGA_16	You wanted to buy organic apples but changed your mind because you were afraid your friends would think you’re overly picky about food. Later you regret it.	**“I pictured them calling me high-maintenance—that thought alone made me hold back.”**	“Their opinion of my food doesn’t matter. I’ll buy organic if I want to.”
VEGA_17	You bought organic cheese but at home were embarrassed to mention it to guests, afraid of being mocked. You feel this is silly.	“I’m comfortable with my choices and don’t need to hide them.”	**“The thought of being mocked for buying something ‘fancy’ made me hide it.”**
VEGA_18	You didn’t buy organic chicken because you were afraid your relatives would think you’re wasting money. Later you regret it.	**“I was mortified they’d think I’m bad with money—so I avoided the situation.”**	“My spending is my business. I’ll buy organic if I want to.”
VEGA_19	You bought regular milk even though you wanted organic, because you were afraid other customers would think you’re a “rich kid.” Later you get angry at yourself.	“I’ll buy what I want regardless of what strangers think.”	**“Felt uneasy that strangers might see me as spoiled—so I settled for regular milk.”**
VEGA_20	You wanted to order an organic salad at a café but changed your mind, afraid that colleagues would think you’re “pretending to be a health nut.” Later you regret it.	**“I hated the thought of them seeing me as a try-hard—so I chose something plain.”**	“I’ll order what I want. Their opinion doesn’t affect my choices.”

Note. **Bold** indicates the narcissistic response. The narcissistic option was coded as 1; the other option was coded as 0 (neutral or healthy). During instrument development, some avoidant items were refined to ensure that the healthy response option was clearly distinguishable from the avoidant response. The original formulations are available upon request. In the original survey, respondents were asked to imagine themselves in each situation and to select the response closest to what they would actually think or do.

## Appendix B

**Table A2:** VN-6 decision-making vignettes (criterion measure).

Item	Situation	Option A	Option B
VN_1	You can receive a $5000 bonus at work. To qualify, you must present your project at a team meeting, after which a colleague will publicly point out a significant mistake you made.	“I’d present the project, take the criticism seriously, and claim the bonus. The feedback is useful, and the money is worth the discomfort.”	**“I’d present the project, take the bonus, and agree publicly. Later I’d tell colleagues the boss was nitpicking—anyone could have made that mistake.”**
VN_2	You made a mistake in an important document you sent to your boss. You can either admit the mistake or stay silent and hope no one notices.	“I’d admit the mistake immediately. Fixing the problem is more important than protecting my ego.”	**“I’d stay silent. If it comes up, I’d say the instructions were unclear—it’s not my fault.”**
VN_3	You’ve tried to get a promotion five times and been passed over each time. A mentor suggests you take a smaller, less prestigious role.	**“I’d apply again. The committee doesn’t recognise talent—they’re threatened by people better than them.”**	“I’d take the smaller role. The pragmatic choice is the smart choice, even if it stings.”
VN_4	You can receive a $1500 bonus at work. To qualify, you must write a brief self-assessment listing three mistakes you made during the year.	**“I’d write the assessment, but focus on ‘lessons learned’ rather than actual mistakes. Why give my manager ammunition?”**	“I’d write the assessment honestly and claim the bonus. Reflecting on mistakes is useful, and the money is real.”
VN_5	You lead a team project. A junior colleague has an idea that would double the project’s success rate, but implementing it would mean they take over the key decisions.	**“I’d take the idea but keep final decisions myself. I’m not letting a junior run things—they lack experience.”**	“I’d hand over the key decisions. Results matter more than who’s in charge.”
VN_6	You’re hiring for a key position. Candidate A is highly competent but questions authority. Candidate B is less competent but fully loyal.	“I’d hire Candidate A. Competence and honesty matter more than blind loyalty to me.”	**“I’d hire Candidate B. Questioning authority is more trouble—I need followers, not thinkers.”**

Note. **Bold** indicates the narcissistic response. In the original survey, respondents were asked to imagine themselves in each situation and to select the response closest to what they would actually think or do.

## Appendix C

Confirmatory Factor Analysis of the VEGA Behavioural Domain

A confirmatory factor analysis (CFA) was performed to test the hypothesised two-factor structure, with items 1–10 assigned to the Antagonistic factor and items 11–20 assigned to the Avoidant factor, following the original instrument design. The analysis used maximum likelihood estimation with weighted adjustments (MLW) in Python (semopy package).

**Table A3:** CFA model fit indices.

Index	Value	Threshold	Interpretation
χ ^2^	278.79	—	—
*df*	169	—	—
χ ^2^ */df*	1.65	<3	Good
CFI	0.83	>0.90	Close to acceptable
TLI	0.81	>0.90	Close to acceptable
RMSEA	0.039	<0.08	Good
GFI	0.66	>0.90	Below threshold
AGFI	0.62	>0.80	Below threshold
NFI	0.66	>0.90	Below threshold

Note. *N* = 422. CFA = confirmatory factor analysis; CFI = comparative fit index; TLI = Tucker–Lewis index; RMSEA = root mean square error of approximation; GFI = goodness-of-fit index; AGFI = adjusted goodness-of-fit index; NFI = normed fit index.

**Table A4:** Standardised factor loadings.

Item	Factor	Std. Loading
VEGA_1	Antagonistic	0.20
VEGA_2	Antagonistic	0.26
VEGA_3	Antagonistic	0.09
VEGA_4	Antagonistic	**0.47**
VEGA_5	Antagonistic	0.08
VEGA_6	Antagonistic	0.11
VEGA_7	Antagonistic	**0.56**
VEGA_8	Antagonistic	0.10
VEGA_9	Antagonistic	0.30
VEGA_10	Antagonistic	**0.59**
VEGA_11	Avoidant	0.22
VEGA_12	Avoidant	0.27
VEGA_13	Avoidant	0.23
VEGA_14	Avoidant	**0.42**
VEGA_15	Avoidant	**0.50**
VEGA_16	Avoidant	**0.61**
VEGA_17	Avoidant	**0.44**
VEGA_18	Avoidant	0.32
VEGA_19	Avoidant	**0.55**
VEGA_20	Avoidant	**0.56**

Note. *N* = 422. Loadings ≥ 0.40 are shown in **bold**. Factor covariance between Antagonistic and Avoidant = 0.28 (*p* = 0.038).

The Avoidant factor showed satisfactory loadings (six items ≥ 0.40), whereas the Antagonistic factor showed weaker loadings (three items ≥ 0.40). The model showed good absolute fit (RMSEA = 0.039; χ^2^*/df* = 1.65) and near-acceptable incremental fit (CFI = 0.83; TLI = 0.81). These results suggest that the two-factor structure is broadly consistent with the data, although the Antagonistic factor requires further refinement through item revision.
